# A personalized 3D printed cranial shield using mirror-image modeling: design and clinical assessment

**DOI:** 10.1186/s41205-025-00289-4

**Published:** 2025-07-01

**Authors:** Zhongjie Shi, Zhengbo Yuan, Jie Chen, Hongwei Zhu, Hualing Huang, Zhanxiang Wang, Zirui Su

**Affiliations:** 1 Department of Neurosurgery, The First Affiliated Hospital of Xiamen University, School of Medicine, Xiamen University, No.55 Zhenhai Road, Siming District, Xiamen, 361001 China; 2 National Institute for Data Science in Health and Medicine, Xiamen University, Xiamen, 361001 China; 3 Xiamen Key Laboratory of Brain Center, Xiamen, 361000 China; 4 Department of Neurosurgery, Xuzhou Central Hospital, Xuzhou, 221000 China

**Keywords:** 3D printing, Decompressive craniectomy, Cranial defect, Personalized

## Abstract

**Background:**

Patients who undergo decompressive craniectomy (DC) are at increased risk of head trauma due to postoperative cranial defects, which not only raise concerns about physical vulnerability but also negatively impact psychological well-being. Conventional protective strategies remain insufficient. This study aimed to develop a personalized, low-cost, three-dimensional (3D) printed external head protection device using mirror-image modeling, and to evaluate its performance in providing physical protection and improving patient-reported outcomes during the post-discharge period.

**Method:**

A prospective study was conducted involving 58 patients treated with DC between August 2023 and February 2025 across two neurosurgical centers. Participants were randomly assigned to an observation group (*n* = 28), who wore a custom-designed 3D printed protective device based on postoperative CT scans, or to a control group (*n* = 30) without special protective measures. A custom questionnaire was used to assess satisfaction with appearance, willingness to engage in social activities, and fear of accidental impact at weeks 1, 4, and 8 post-discharge. Objective indicators such as fall events, adverse reactions, and device integrity were also recorded.

**Results:**

The 3D printed models demonstrated good anatomical fit and structural reliability. At weeks 4 and 8, the observation group showed significantly higher Visual Analog Scale (VAS) scores compared to the control group (*P* = 0.014 and *P* = 0.002, respectively), with continuous improvement over time (*P* < 0.05). The average daily usage time of the device was 4.4 ± 1.2 h. No cases of skin irritation or pressure injuries were reported. One patient in the observation group experienced a fall that caused a minor device crack but no head injury (fall rate: 3.6%). In the control group, two patients fell without head trauma (fall rate: 6.7%).

**Conclusions:**

Our findings introduce a personalized, 3D printed external helmet as a new option for cranial protection after decompressive craniectomy. The prototype provided reliable mechanical shielding, conformed closely to each patient’s skull contour, and was well tolerated. By reducing physical risk, boosting confidence in appearance, and alleviating anxiety during the interval before cranioplasty, the device may bridge a critical safety and psycho-social gap in early rehabilitation.

**Supplementary Information:**

The online version contains supplementary material available at 10.1186/s41205-025-00289-4.

## Introduction

Cranial defects, arising from a range of etiologies, are a common challenge in neurosurgical practice [1, 2]. The majority of these defects result from DC, a procedure typically performed to alleviate intracranial pressure, restore cerebral perfusion, and prevent irreversible brain damage, thereby saving the patient’s life [3–6]. DC is primarily indicated for patients with elevated intracranial pressure due to traumatic brain injury, intracerebral hemorrhage, large cerebral infarction, brain edema, or skull fractures.

Patients usually undergo a secondary surgical procedure to repair the cranial defect within 3 to 6 months after DC [7–9]. During this time, the absence of bony support leaves the scalp vulnerable, making the brain susceptible to secondary injury caused by external impact or falls. This risk is particularly pronounced in the early postoperative period, when patients often experience impaired balance and coordination, further increasing their fall risk. Moreover, the visible cranial deformity caused by bone loss can significantly affect patients’ appearance, potentially leading to psychological issues such as body dysmorphic disorder (BDD) and social anxiety disorder (SAD), which negatively impact quality of life [10, 11]. Therefore, providing appropriate cranial protection before cranioplasty is not only critical for ensuring physical safety but also essential for supporting psychological recovery.

In clinical practice, protecting the cranial defect site is a key aspect of postoperative care for DC patients. While hospitalized, patients benefit from consistent monitoring by a professional nursing team, and serious complications caused by cranial defects are relatively rare [12, 13]. However, as recovery progresses and patients are discharged, the absence of continued professional care increases the risk of accidental injury, which may result in rehospitalization, adding to both clinical risk and financial burden [14]. Consequently, personalized and sustained protection of the cranial defect area is an essential component of post-DC neurosurgical nursing care. Current literature [15, 16] reports that available external protective devices for cranial defects often lack individual customization, fail to ensure comfort, and are aesthetically suboptimal.

In this study, we utilized digital imaging-based 3D reconstruction and thin-slice CT data to develop a personalized head protective device (PHPD) for post-DC patients. By employing mirrored modeling of the scalp, the device was designed to be lightweight, aesthetically pleasing, and cost-effective, offering effective and continuous protection of the cranial defect area [17]. It is expected that the PHPD will enhance the physical safety of the defect site, improve patients’ self-image and psychological well-being, and ultimately promote holistic recovery.

## Methods

### Patient selection

**Fig. 1:**
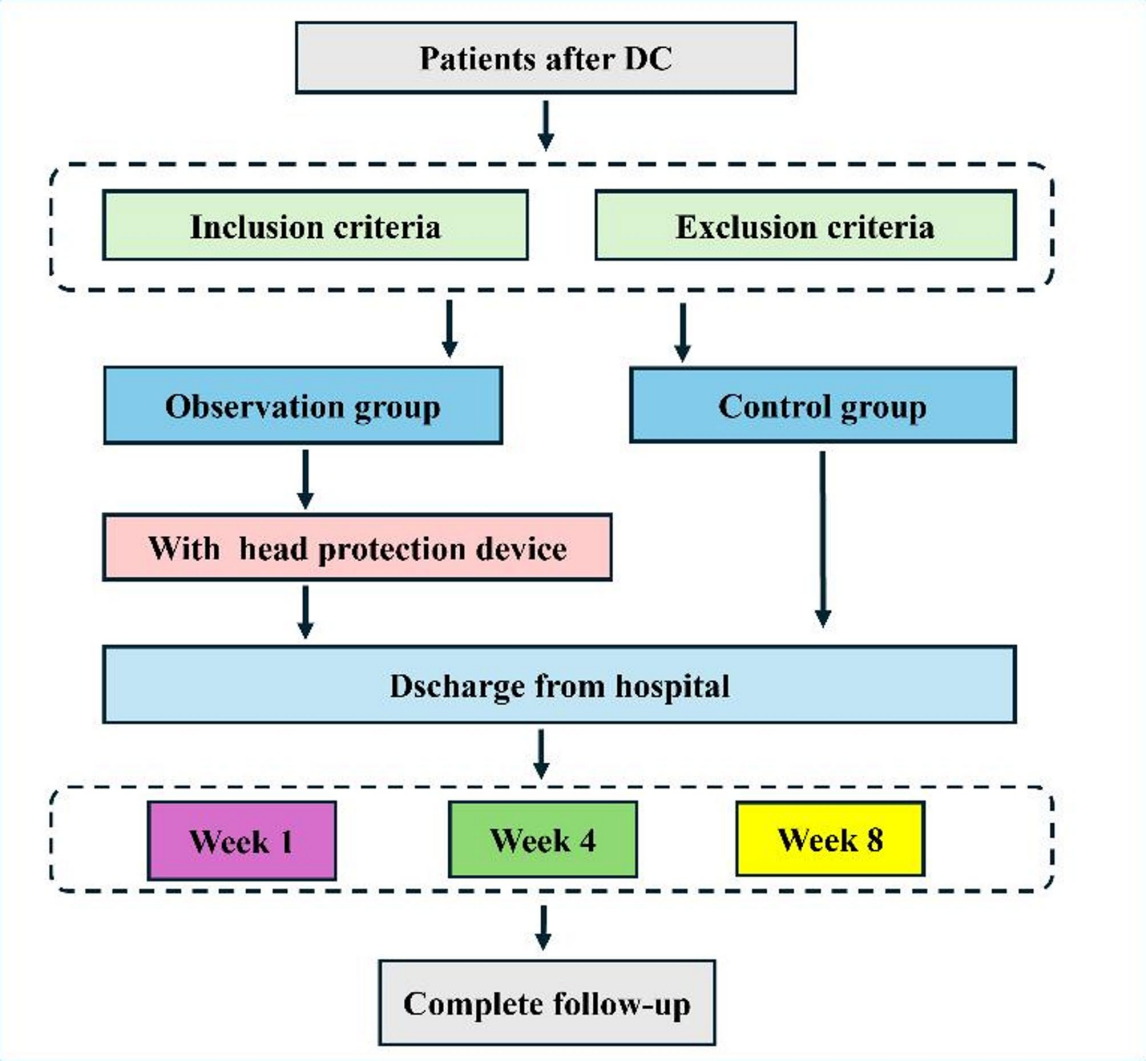
Patient enrollment and study flow diagram

This study prospectively enrolled patients who underwent their first DC in the neurosurgery departments of two hospitals in China between August 2023 and February 2025. Eligible patients were randomly assigned to either the observation group (PHPD group) or the control group (routine care group) according to a computer-generated randomization sequence. Patients in the observation group were provided with a customized head protective device upon discharge, while those in the control group were instructed to follow standard discharge guidance and their usual lifestyle practices. All patients were followed for two months post-discharge (Fig. 1).

This study was conducted in accordance with the ethical principles of the Declaration of Helsinki and was approved by the Clinical Research Ethics Committee of the First Affiliated Hospital of Xiamen University and Xuzhou Central Hospital.

**Inclusion Criteria**:

- Age ≥ 18 years, with stable vital signs postoperatively, well-healed incision, and suture removal completed;
- Clear consciousness at discharge, with the ability to accurately express subjective feelings and independently complete evaluation scales;
- Maximum diameter of the cranial defect (cross-sectional long axis) > 3 cm;
- Voluntary agreement to use the protective device, with informed consent signed by the patient and/or their legal representative during hospitalization.

**Exclusion Criteria**:

- Postoperative intracranial or incision infection;
- Comatose or unconscious state at discharge;
- Severe dysfunction of vital organs (heart, liver, kidney, or lungs);
- Presence of severe psychiatric disorders.

### Imaging acquisition and 3D modeling

The 3D modeling software used in this study was 3D Slicer (Version 5.6, www.slicer.org), an open-source, cross-platform software platform for medical image analysis and 3D visualization [18]. Developed at Harvard Medical School, Brigham and Women’s Hospital and Harvard Medical School, and modified by other institutions. 3D Slicer is widely applied in neurosurgery, radiology, oncology, and image-guided therapy [19–21].

All patients underwent thin-slice cranial CT scans (Philips Brilliance 64) three days prior to discharge. The scanning parameters were as follows: slice thickness of 1 mm, continuous acquisition, tube voltage of 120 kV, tube current of 200–250 mA, matrix size of 512 × 512, and field of view (FOV) of 220 mm. The CT images were exported from the hospital picture archiving and communication system (PACS) in digital imaging and communications in medicine (DICOM) format and anonymized prior to processing.

**Fig. 2:**
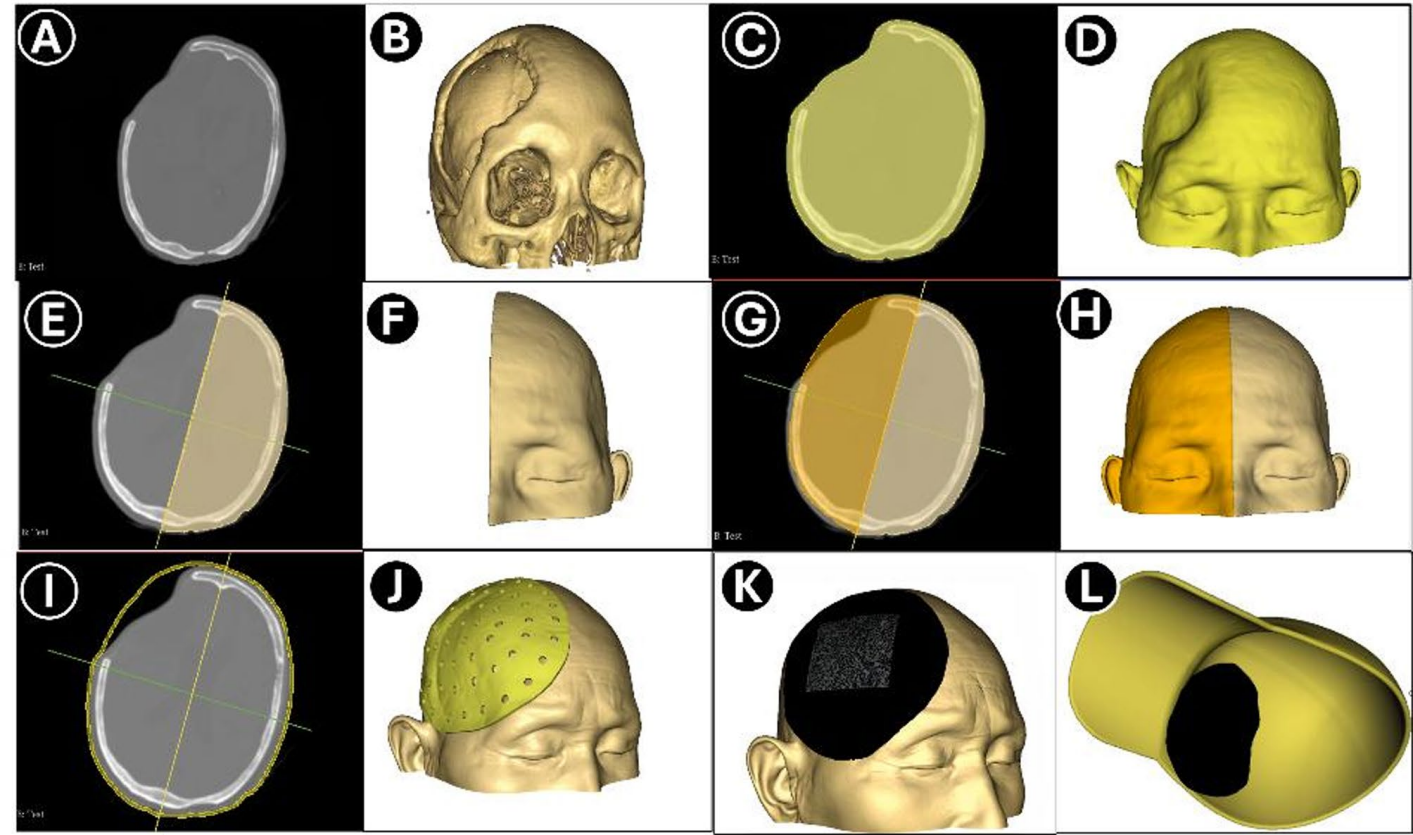
Design workflow of the PHPD. **A**–**B**: Original CT images and skull 3D reconstruction; **C**–**D**: Segmentation of the head region using thresholding to generate a solid model; **E**–**F**: Trimming along the mid-sagittal plane to retain the unaffected side; **G**–**H**: Mirroring the preserved side to reconstruct a complete head model; **I**–**J**: Expanding the model outward by 2.5 mm, hollowing the interior, and trimming to form the final device shape; **K**–**L**: Wrapped the device in soft black fabric and secured it inside a daily-use cap using nylon fasteners

The images were then imported into 3D Slicer. In the “*Segment Editor*” module, a new segment was created. Using the “*Threshold*” tool, an appropriate intensity range was selected to segment the entire head structure, including scalp, skull, and brain tissue, generating a complete head model. The median sagittal plane was then marked using the “*Interaction*” tool. Based on this plane, the “*Mirror*” module was applied to create a mirrored reconstruction of the contralateral (healthy) side. The mirrored surface was manually adjusted to best match the defect area. Using the “*Scissors*” tool, the model was trimmed around the scalp depression, ensuring the protective area extended at least 2.5 cm beyond the margins of the skull defect. To improve breathability, 5 mm-diameter ventilation holes were designed across the surface of the model (Fig. 2). The final design was exported in STL format and 3D printed using polylactic acid (PLA) material (YUG, China). We have provided a detailed fabrication tutorial in the supplementary materials.

**Fig. 3:**
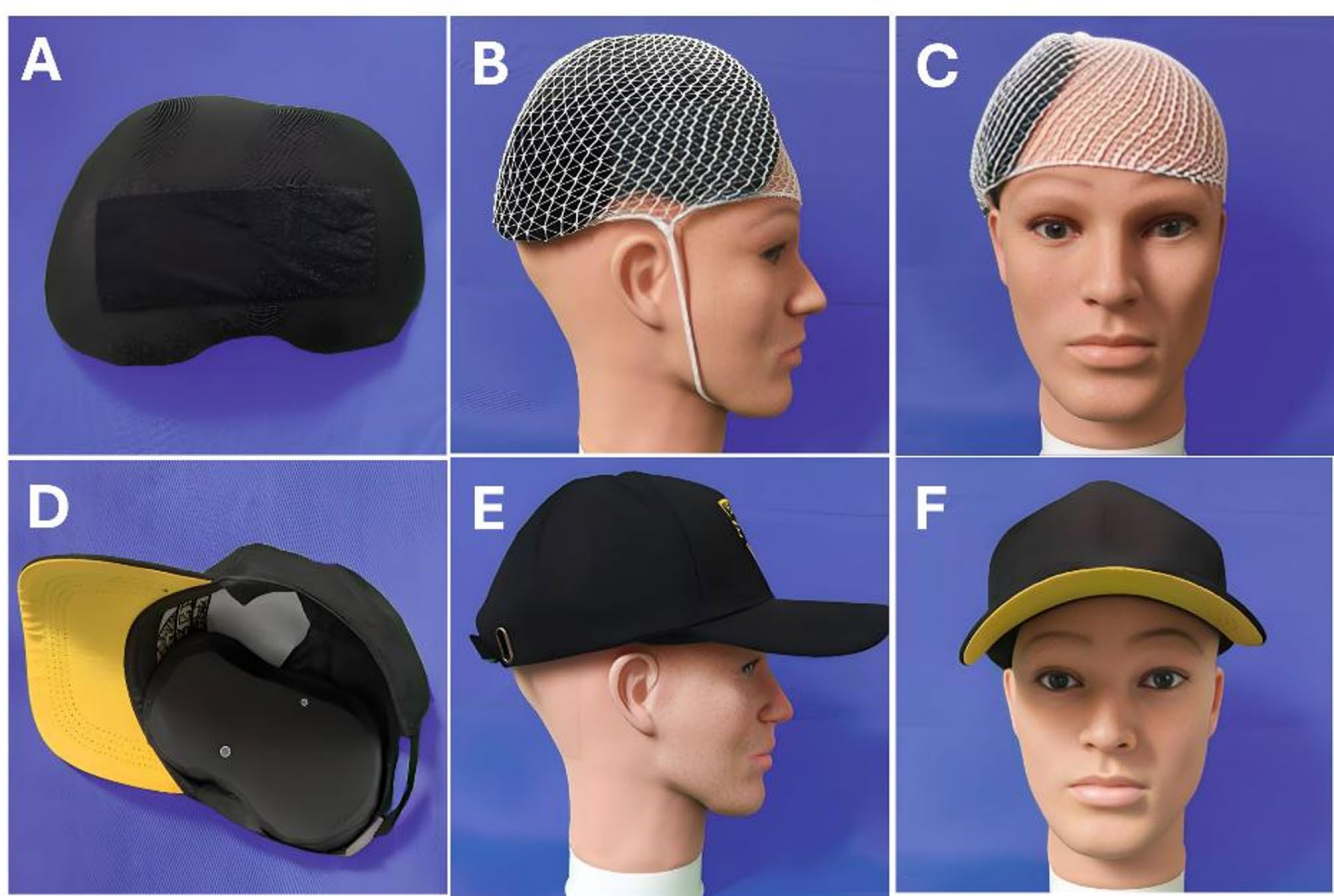
Packaging and fixation of PHPD. **A**: Fabricated using 3D printing and covered with black breathable fabric; **B**–**C**: A medical elastic mesh cap was used to secure the device to the head; **D**–**F**: Nylon hook-and-loop fasteners were used to attach the device to a regular daily cap, ensuring stable fixation and convenient use

The printed device was covered with breathable black fabric, and nylon fasteners were attached to its outer surface. Based on patient and caregiver preferences, different types of daily wear hats (e.g., baseball caps, berets, flat caps) were selected. Matching nylon fasteners were affixed to the inner surface of the hats at corresponding positions, allowing the protective device to be securely attached and worn comfortably (Fig. 3).

### Instructions for use of PHPD

After wound healing and removal of scalp sutures, the attending physician evaluated the patient’s surgical site for signs of redness, exudation, or other abnormalities to determine eligibility for device application. For eligible patients, the attending nurse assisted with fitting the protective device and assessed whether the device’s edge conformed adequately to the scalp contour. The device was initially secured using a medical elastic cap and worn for 0.5 h under supervision. Skin around the edge of the device was observed for impressions or discomfort. If no abnormalities were noted, patients continued to wear the device with hourly monitoring. The patient’s subjective comfort was assessed during each interval. In the event of discomfort, intolerance, or local skin reactions such as erythema or swelling, device use was immediately discontinued.

After discharge, patients could replace the elastic cap with a pre-selected daily-use hat. Before discharge, the attending nurse educated the patient and caregivers on key precautions for device use: (1) Ensure the patient fully understands the correct method of use; (2) Inspect the device for structural damage or deformation before each use and monitor for skin irritation after use; (3) Report any adverse events such as falls or structural failure of the device promptly; (4) Maintain a daily log of the device wearing time.

### Evaluation indicators

The effectiveness of PHPD was evaluated across both **subjective** and **objective**:

Subjective evaluation indicators were measured using a self-designed satisfaction questionnaire based on clinical practice, with ratings quantified via the VAS. The questionnaire assessed five dimensions, each scored from 1 to 10 (1 = extremely dissatisfied/worried, 10 = extremely satisfied/no concern), with a total score range of 5 to 50:

- Satisfaction with head appearance;
- Confidence in head stability during daily activities;
- Willingness to engage in social interactions;
- Concern about accidental head impacts (reverse scored);
- Overall satisfaction with postoperative head protection.

Questionnaires were distributed electronically via WeChat mini-programs and email at the 1st, 4th, and 8th week after discharge.

Objective evaluation indicators included the device’s weight, occurrence of falls during follow-up, structural integrity of the device, and adverse events such as skin indentations, localized pain, or itching. All outcomes were documented by clinical nursing staff or reported by the patients during the follow-up period.

### Sample size estimation

The sample size was determined based on the expected clinical case volume at the participating centers. According to clinical experience, an expected mean difference of 5 points (SD = 6) in total VAS scores between groups was assumed, corresponding to an effect size (Cohen’s d) of approximately 0.83. With a two-sided test, significance level α = 0.05, power = 0.80, and equal group sizes, the minimum sample size required was calculated as 24 participants per group, totaling 48. Considering an estimated 5% attrition rate, 25 participants per group (*n* = 50 total) were ultimately required.

Data collection followed a standardized protocol using predesigned case report forms, and all data were gathered by trained personnel under unified procedures. This study design also adhered to the **GRACE (Good Research for Comparative Effectiveness)** principles (https://www.graceprinciples.com/index.html) and followed the **GCP (Good Clinical Practice)** guidelines (https://www.ema.europa.eu/en/ich-e6-good-clinical-practice-scientific-guideline) to ensure data integrity and reporting transparency (cf. Heller R, Krieger A, Rosset S. *Optimal multiple testing and design in clinical trials.* Biometrics. 2023;79(3):1908–1919).

### Statistical analysis

All data were analyzed using SPSS version 26.0 (SPSS Inc., Chicago, IL, USA). Categorical variables (e.g., device breakage, incidence of adverse events) were expressed as frequencies (n) and percentages (%) and compared using the Chi-square test or Fisher’s exact test when expected counts were low. Continuous variables were expressed as mean ± standard deviation (SD). Between-group comparisons were conducted using the Mann–Whitney U test, and within-group comparisons across different time points were analyzed using the Wilcoxon signed-rank test. All tests were two-tailed, and a *P* value < 0.05 was considered statistically significant.

## Results

In this study, a total of 29 personalized external head protective devices were successfully designed by clinical physicians based on digital 3D reconstruction of patients’ cranial defect regions. The initial fit between the PHPD and the edge of the scalp surrounding the cranial defect was satisfactory in all cases, and no adverse reactions were observed during the first trial use.

### Baseline characteristics of the patients

A total of 59 patients were enrolled in this study. Among them, 29 patients were initially assigned to the observation group. One patient withdrew during follow-up, resulting in 28 patients included in the final analysis (15 males and 13 females), with a mean age of 47.5 ± 16.7 years (range: 22–78 years).

**Fig. 4:**
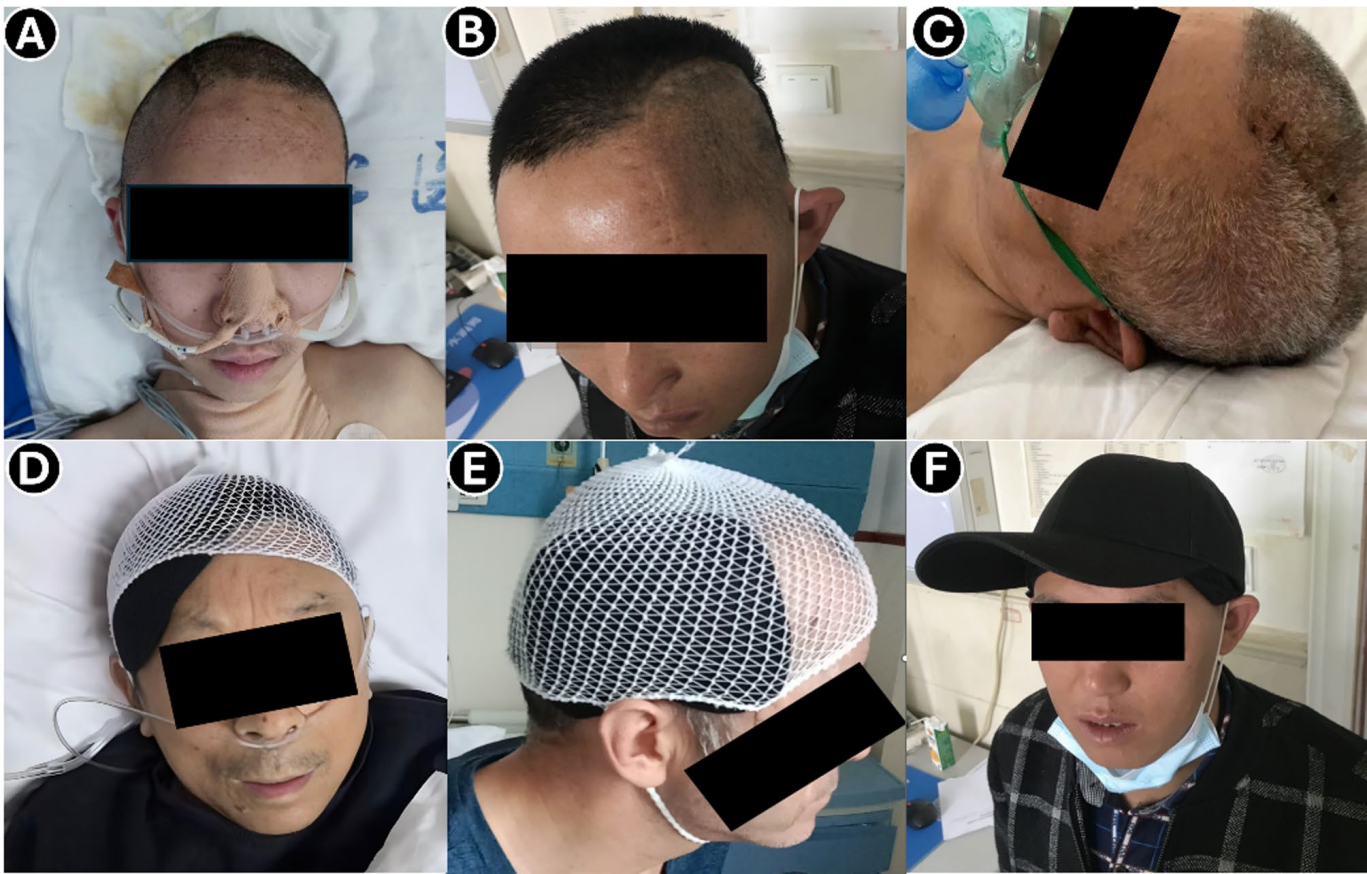
Postoperative appearance and use of PHPD. **A**–**C**: Depressed, flat, and elevated cranial profiles observed after DC due to intracranial pressure differences; **D**–**E**: Device secured during hospitalization using elastic mesh caps; **F**: Device fixed inside a daily-use cap for use in everyday life

The average transverse diameter of the cranial defects in the observation group was 10.8 ± 2.3 cm, with a median of 10.9 cm (range: 5.7–14.9 cm), and a 95% confidence interval of [9.90, 11.71]. Fig. 4 illustrates the cranial morphology of a typical post-DC patient and appearance after wearing the protective device.

**The control group** included 30 patients (14 males and 16 females), with a mean age of 50.1 ± 20.2 years (range: 19–80 years). The average transverse diameter of the cranial defect in the control group was 10.3 ± 2.5 cm, with a median of 10.8 cm (range: 5.8–14.5 cm) and a 95% confidence interval of [9.37, 11.20].

- Age comparison between groups using the Mann–Whitney U test showed no significant difference (U = 384, *p* = 0.58);
- Gender distribution comparison using the Chi-square test also showed no significant difference (χ² = 0.07, *p* = 0.80);
- Comparison of cranial defect diameters using the Mann–Whitney U test revealed no significant difference (U = 460, *p* = 0.54).

There were no statistically significant differences in demographic characteristics or cranial defect size between the two groups **(**Table 1**)**.

**Table 1:** Baseline characteristics and follow-up outcomes of all patients

Variable	Observation Group	Control Group	Test Statistic/Test Method	*P*-Value
**Baseline Characteristics**				
Sex (Male/Female)	15 /13	14 / 16	χ² = 0.07	0.800
Age (years)	47.5 ± 16.7	50.1 ± 20.2	U = 384	0.580
Defect Diameter (cm)	10.8 ± 2.3	10.3 ± 2.5	U = 460	0.540
Weight of Device(g)	71.8 ± 6.5	—	—	—
Daily Duration of Wearing (h/day)	4.4 ± 1.2	—	—	—
Fall Events (cases)	1 / 28(3.6%)	2 / 30(6.7%)	Fisher Test	1.000
**Between-Group Comparison**				
VAS Score, Week 1	29.9 ± 4.4	29.5 ± 5.1	U = 436	0.809
VAS Score, Week 4	32.5 ± 5.0	29.6 ± 4.4	U = 579	0.014
VAS Score, Week 8	35.8 ± 5.7	30.8 ± 5.1	U = 621	0.002
**Within-Group Comparison**	**Observation Group**		**Control Group**	
Week 1 vs. Week 4	W = 58.5	*p* = 0.003	W = 63.0	*p* = 0.796
Week 4 vs. Week 8	W = 43.0	*p* < 0.001	W = 53.0	*p* = 0.436

Note: VAS = Visual Analog Scale; U = Mann–Whitney U test; χ² = Chi-square test

### VAS score evaluation

**Fig. 5:**
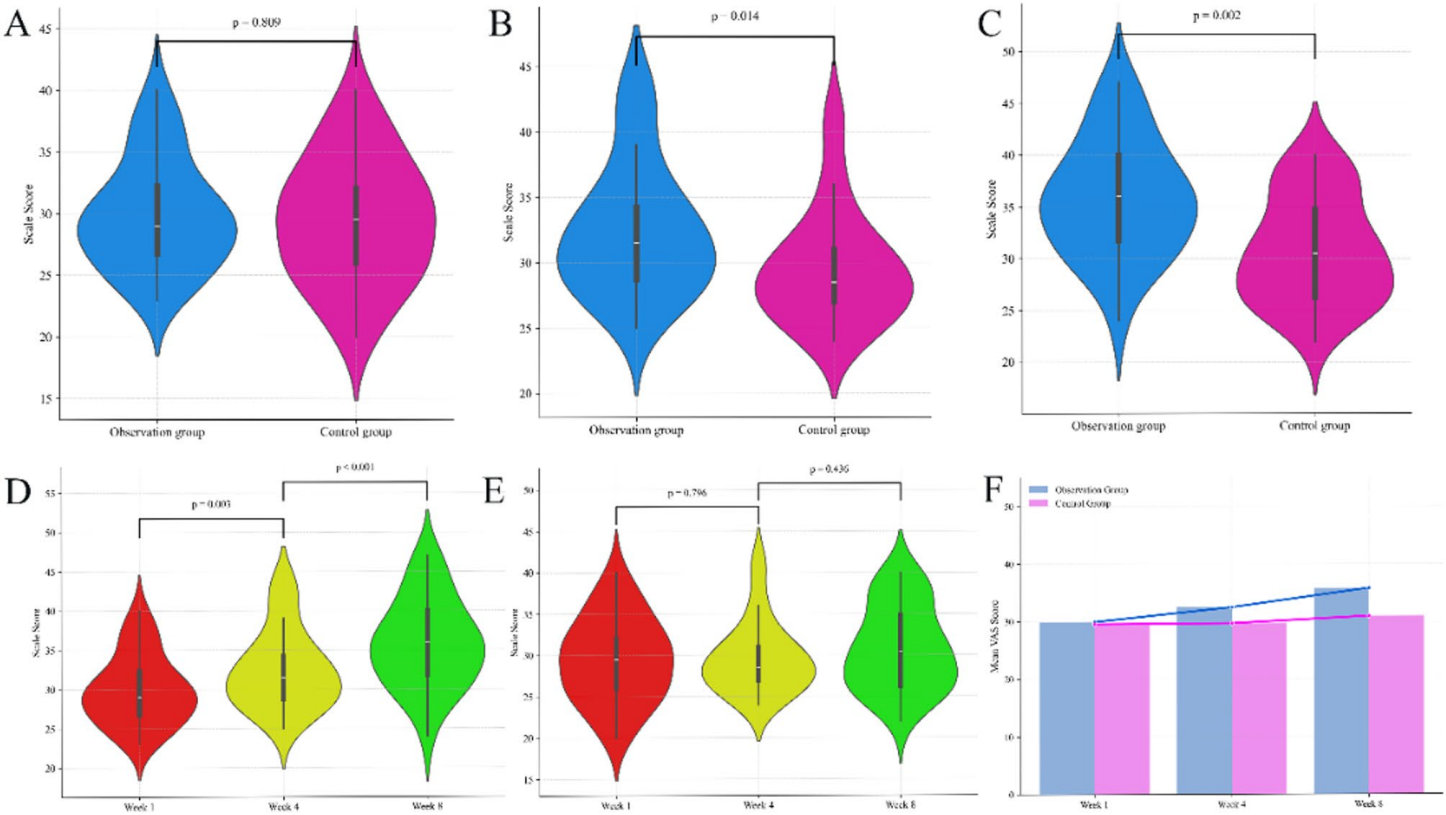
Analysis of VAS. **A**–**C**: Violin plots comparing VAS scores between the observation and control groups at Weeks 1, 4, and 8, respectively. No significant difference was observed at week 1, while significant differences emerged at weeks 4 and 8. **D**–**E**: Line plots showing VAS scores across the three follow-up time points within each group. The observation group exhibited a gradual increase in scores over time, whereas the control group showed no notable change. **F**: Bar chart comparing VAS scores with the observation group showing progressively higher scores than the control group

In the first week of follow-up, the observation group had a mean VAS score of 29.9 ± 4.4 (95% CI: [28.21, 31.64]), with a median of 29.0 and a range of 23–40. The control group had a mean score of 29.5 ± 5.1 (95% CI: [27.55, 31.38]), with a median of 29.5 and a range of 20–40. The difference was not statistically significant (Mann–Whitney U = 436, *p* = 0.809) (Fig. 5A).

At week 4, the observation group had a mean score of 32.5 ± 5.0 (95% CI: [30.52, 34.41]), median 31.5, range 25–43, while the control group scored 29.6 ± 4.4 (95% CI: [27.98, 31.22]), median 28.5, range 24–41. The difference was statistically significant (Mann–Whitney U = 579, *p* = 0.014) (Fig. 5B), indicating higher scores in the observation group.

At week 8, the observation group showed a further increase in mean score to 35.8 ± 5.7 (95% CI: [33.59, 37.98]), with a median of 36.0 and range 24–47, while the control group had a mean score of 30.8 ± 5.1 (95% CI: [28.86, 32.67]), median 30.5, range 22–40. The difference remained statistically significant (Mann–Whitney U = 621, *p* = 0.002) (Fig. 5C), again favoring the observation group.

### Longitudinal changes in VAS scores

Wilcoxon signed-rank tests were used to compare changes within each group over time. In the observation group, a significant improvement was observed between week 1 and week 4 (W = 58.5, *p* = 0.003), as well as between week 4 and week 8 (W = 43.0, *p* < 0.001) (Fig. 5D). In contrast, the control group showed no significant change between week 1 and week 4 (W = 63.0, *p* = 0.796), nor between week 4 and week 8 (W = 53.0, *p* = 0.436) Fig. 5E. These results suggest a gradual upward trend in VAS scores over time in the observation group Fig. 5F.

### Objective evaluation indicators

In this study, the average weight of a single protective device was 71.8 ± 6.5 g (95% CI: [69.22, 74.28]), with a median of 70.5 g and a range of 59.0–87.0 g. According to the records of patients in the observation group, the average daily usage duration of the protective device was 4.4 ± 1.2 h (95% CI: [3.91, 4.81]), with a median of 4.3 h and a range of 2.2–6.2 h.

During the follow-up period, no cases of skin erythema, pruritus, or other discomfort were reported, reflecting the safety of the device. Additionally, no mechanical pressure injuries occurred; only minor indentation marks were observed, suggesting the device’s reliability and suitability for long-term wear.

One patient in the observation group experienced a fall during follow-up, resulting in a cracked device after impact with a wall. The incidence of unexpected events in the observation group was 3.6% (1/28). A replacement device was provided, and importantly, despite the fall and damage to the protective device, no secondary head injury occurred, indicating a potential cushioning effect of the device during impact.

In the control group, two patients experienced accidental falls during the follow-up period, yielding an incidence of 6.7% (2/30); however, neither resulted in head contact or secondary injury. Fisher’s exact test showed no significant difference in the incidence of falls between the two groups (*p* = 1.00).

## Discussion

DC is a widely used neurosurgical procedure for controlling intracranial hypertension caused by traumatic brain injury, intracerebral hemorrhage, and other etiologies. While DC has been shown to be effective in reducing intracranial pressure and improving prognosis, postoperative patients remain at risk for various complications [3, 22, 23]. In addition to the physiological risks associated with primary brain injury, cranial deformities due to bone defects can lead to significant psychological distress. Moreover, compared to the general population, DC patients may have a higher risk of falling. It is estimated that approximately one-third of individuals aged ≥ 65 years experience falls annually, and this proportion rises to nearly 50% among those aged ≥ 80 years [24]. Although studies specifically reporting severe fall-related outcomes in DC patients are limited—possibly due to underreporting or the rarity of such events—when these incidents do occur, they are often fatal. Honeybul et al. [25] documented a case in which a patient with satisfactory postoperative recovery died following a fall that directly impacted the unprotected surgical site, highlighting the need for vigilant prevention and management of such events.

### Advantages of the customized head protection device

In this study, we designed a customized head protection device that precisely conforms to the defect site using mirrored modeling of the scalp surface. The preliminary results suggest that the device may effectively cushion impact forces and reduce the risk of secondary injury, while also enhancing patients’ confidence in daily activities, indicating its potential for clinical application.

Regarding material selection, we chose polylactic acid (PLA), a biocompatible and biodegradable material synthesized from renewable plant-based resources. PLA is non-irritating to the skin and is considered safe for use in sensitive populations such as the elderly and children [26, 27]. For the headgear structure, we adopted an adjustable daily-use cap, which offers easy removal, portability, and flexibility to suit different daily scenarios. The overall production cost, including 3D printing materials and packaging, was kept under USD 30, making it affordable for low- and middle-income families and feasible for wide adoption across different levels of healthcare institutions.

It is worth noting that our device design is based on the patient’s actual scalp contour reconstructed in 3D. This approach is applicable to various types of cranial defects. Compared to previous studies that designed protection devices based on skull extension models [28, 29], our method better conforms to the true soft tissue morphology—especially in the temporal region, where the temporalis muscle is thicker than in other areas of the head [30, 31]. Designs solely based on cranial bone extension may lead to poor fit, whereas scalp contour modeling provides better anatomical accuracy, improving both comfort and aesthetics.

### Preliminary evaluation of device use

Our findings revealed that patients used the device for an average of 5.6 ± 1.4 h per day, suggesting selective rather than continuous use, aligned with individual needs and further supporting the necessity for a portable design. At week 1 of follow-up, no statistically significant difference was observed in life satisfaction (VAS score) between the intervention and control groups (29.9 ± 4.4 vs. 29.5 ± 5.1, *p* = 0.809), possibly due to the short adaptation period after surgery. However, at weeks 4 and 8, the observation group exhibited significantly higher VAS scores compared to the control group (Week 4: 32.5 ± 5.0 vs. 29.6 ± 4.4, *p* = 0.014; Week 8: 35.8 ± 5.7 vs. 30.8 ± 5.1, *p* = 0.002), suggesting that the positive impact of the device on patient confidence increased over time.

Importantly, one case of device fracture occurred in the observation group due to a fall (3.6%), but no secondary head injury was reported, suggesting potential protective capability of the device upon impact. In the control group, two patients experienced falls (6.7%), although no head injury occurred.

### Limitations

This study has several limitations. First, the small sample size and dual-center design limit the generalizability and statistical power of the findings. Due to the low incidence of fall-related head injuries, the device’s real-world protective efficacy during actual falls remains difficult to verify. Second, the follow-up duration was relatively short, which prevents evaluation of long-term safety and durability. Third, patients’ socioeconomic and environmental factors (e.g., urban vs. rural residence, living space) were not fully considered, potentially affecting compliance and subjective experience. Finally, some outcome measures relied on self-reported data, which may introduce bias.

Future studies should address these limitations through: (1) multicenter prospective trials with larger sample sizes to validate real-world protective performance during falls; (2) extended follow-up to assess long-term safety, durability, and impact on quality of life; (3) continuous optimization of modeling and printing techniques to improve fit and comfort, and the exploration of lighter, more breathable medical-grade materials; and (4) the incorporation of multisource feedback—including nursing staff, caregivers, and wearable device data—to enhance objectivity and clinical relevance.

## Conclusion

In this study, we proposed a customized 3D printed head protection device based on mirrored modeling of the scalp surface and validated its feasibility in patients undergoing DC. Our results demonstrated favorable safety, comfort, and patient acceptability. Participants wearing the device showed significantly improved life satisfaction at 4 and 8 weeks postoperatively, with no reported complications. Preliminary evidence suggests the device may provide buffering and protective effects against unintentional impact, highlighting its potential role in postoperative rehabilitation and social reintegration. Further evaluation is warranted.

## Electronic supplementary material

Below is the link to the electronic supplementary material.

Supplementary Material 1

## Data Availability

All the data analyzed during this study are included in the article. A detailed fabrication protocol of the personalized head protective device (PHPD) is provided in the supplementary materials.

## Abbreviations

DC: Decompressive Craniectomy
3D: Three-Dimensional
PHPD: Personalized Head Protective Device
VAS: Visual Analog Scale
BDD: Body Dysmorphic Disorder
SAD: Social Anxiety Disorder
FOV: Field of View
PACS: Picture Archiving and Communication System
DICOM: Digital Imaging and Communications in Medicine
PLA: Polylactic Acid
