# Supplementary material for: A personalized 3D printed cranial shield using mirror-image modeling: design and clinical assessment
Source: 3D Print Med. 2025 Jul 1;11:32. doi: 10.1186/s41205-025-00289-4 (PMC12210813; doi:10.1186/s41205-025-00289-4)

Step-by-Step Guide to Generating Personalized Head Protective Device (PHPD) Models with 3D Slicer

Note 1: This protocol demonstrates one design approach using 3D Slicer as the demonstration platform. If you are proficient with other medical image processing software, such as ITK-SNAP, you can also apply this method accordingly.

Note 2: Before beginning, please ensure that 3D Slicer is properly installed and that you have thin-slice CT data available in DICOM format. The main modules utilized in this protocol are Segment Editor, Dynamic Modeler, Data, and Volume Rendering, with Segment Editor serving as the core tool. This document provides a concise operational guide. In practice, adjustments may be required based on the specific characteristics of the imaging data to achieve an optimal personalized model.

1. Program Download and Installation: Download and Install the Latest Version of 3D Slicer（https://download.slicer.org）. If you are new to 3D Slicer, we recommend taking some time to explore its basic features. The official website offers comprehensive resources, and there is an active community (https://discourse.slicer.org/) available to assist with any questions or challenges .

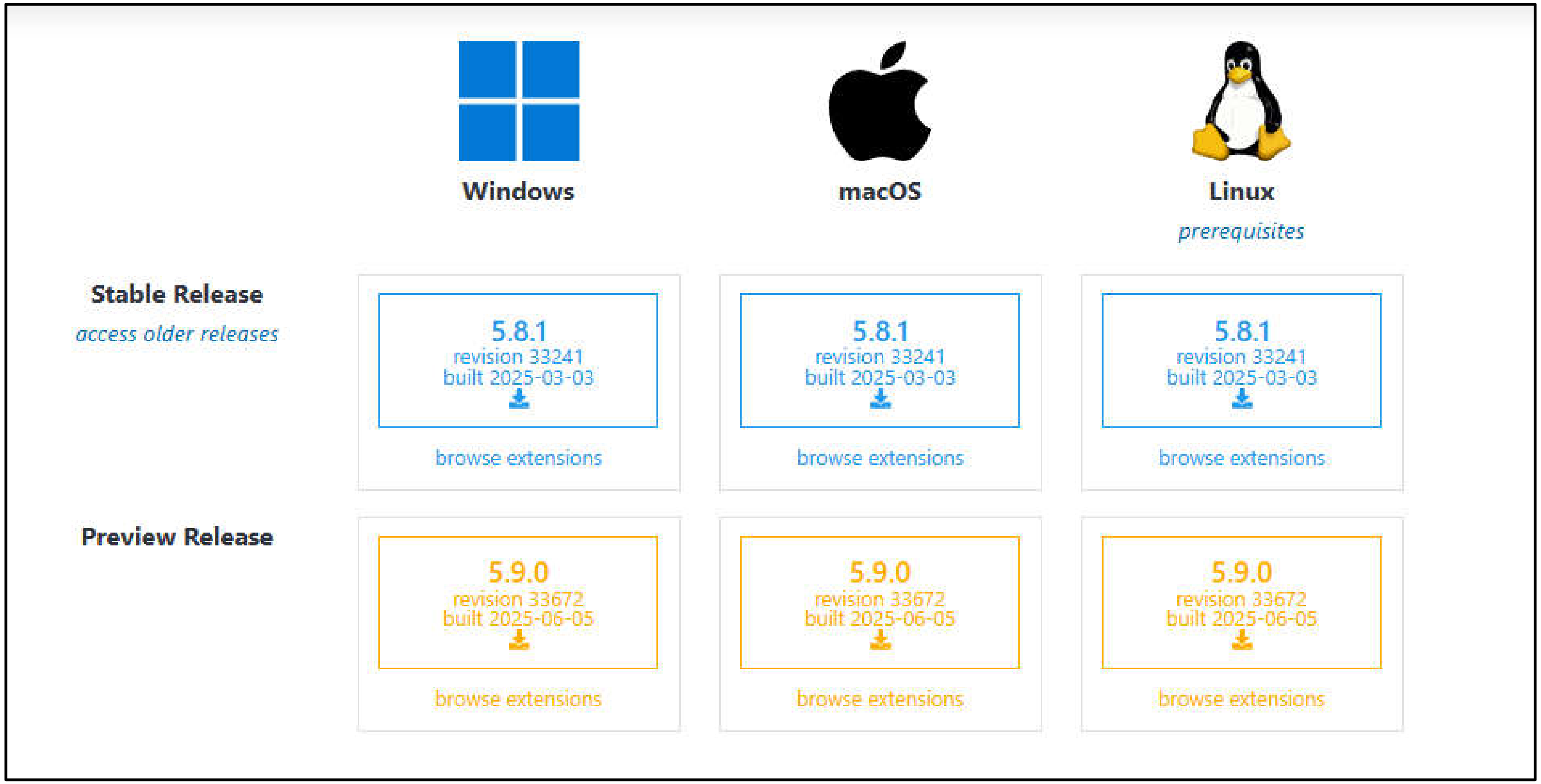

2. Data Loading: The imaging format is DICOM. Simply drag and drop the folder containing the images into the Slicer interface to automatically read the data. From the Data module, select the image series intended for 3D modeling, then click *Load* to import the data.

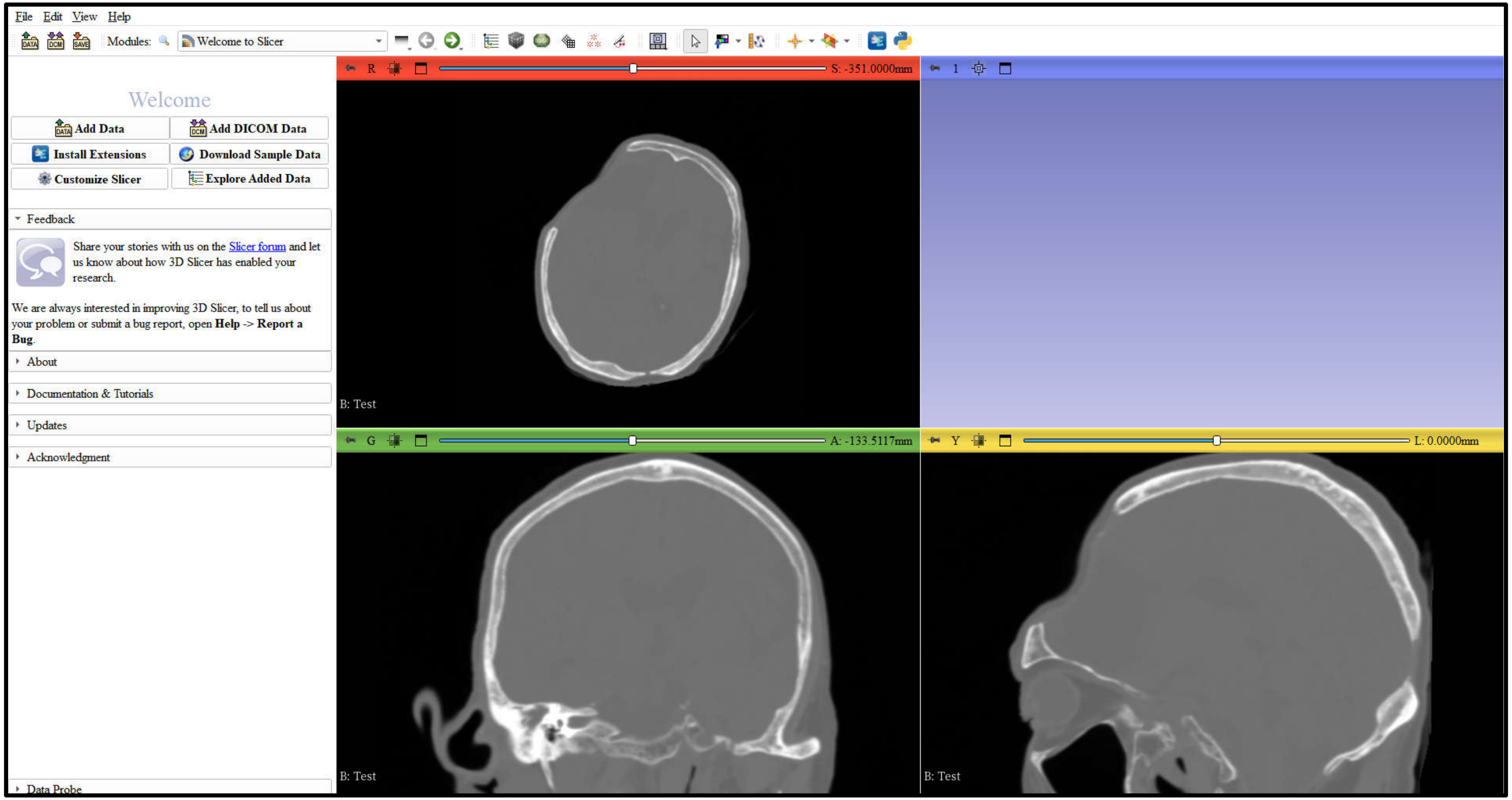

3. Model Creation: In the toolbar, select the *Segment Editor* module, then click *Add* to create a new segment.

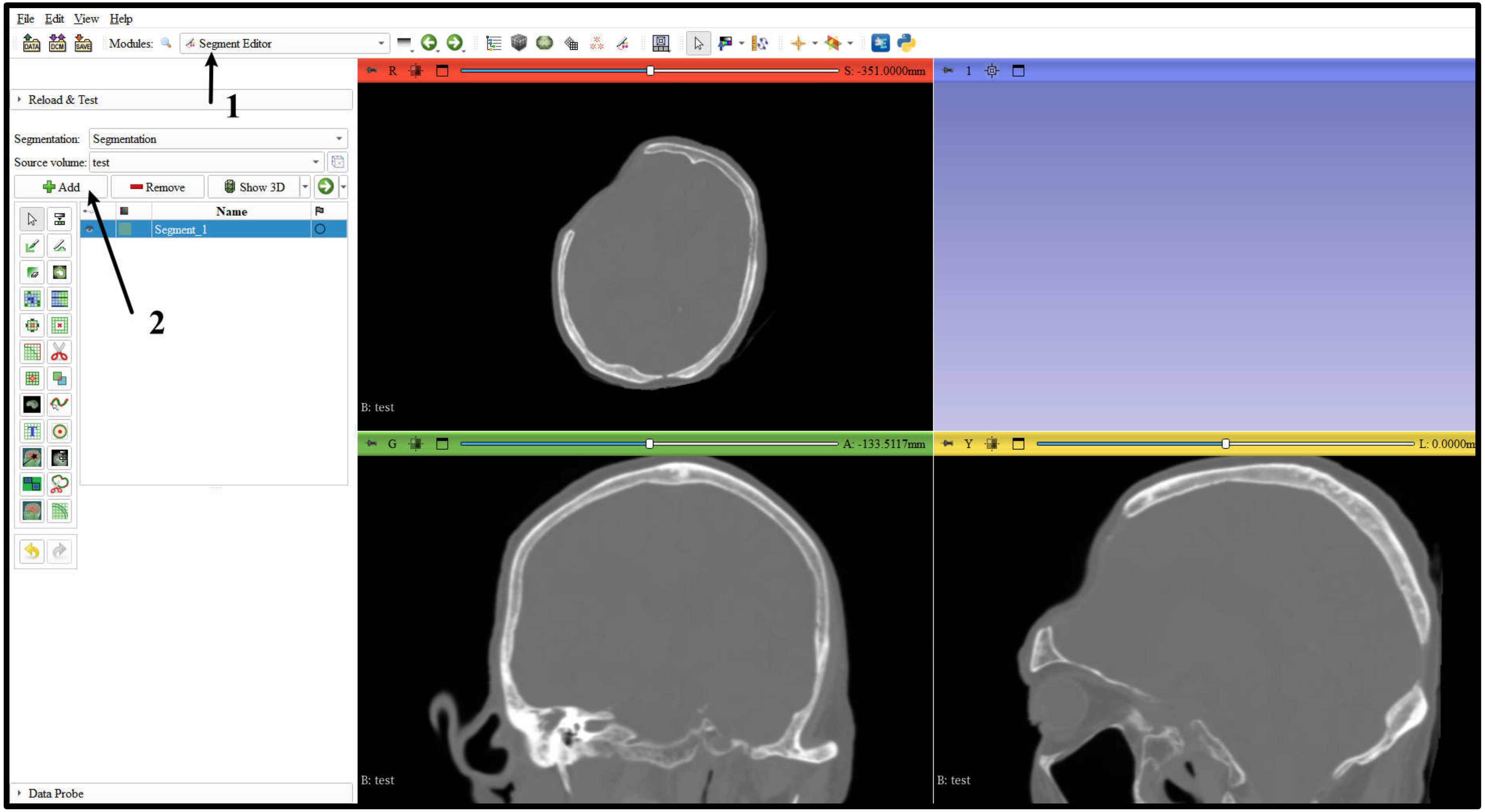

4. Threshold Tool: Select the *Threshold* effect, adjust the threshold range until the entire head is selected, then click *Apply* to generate a complete head model.

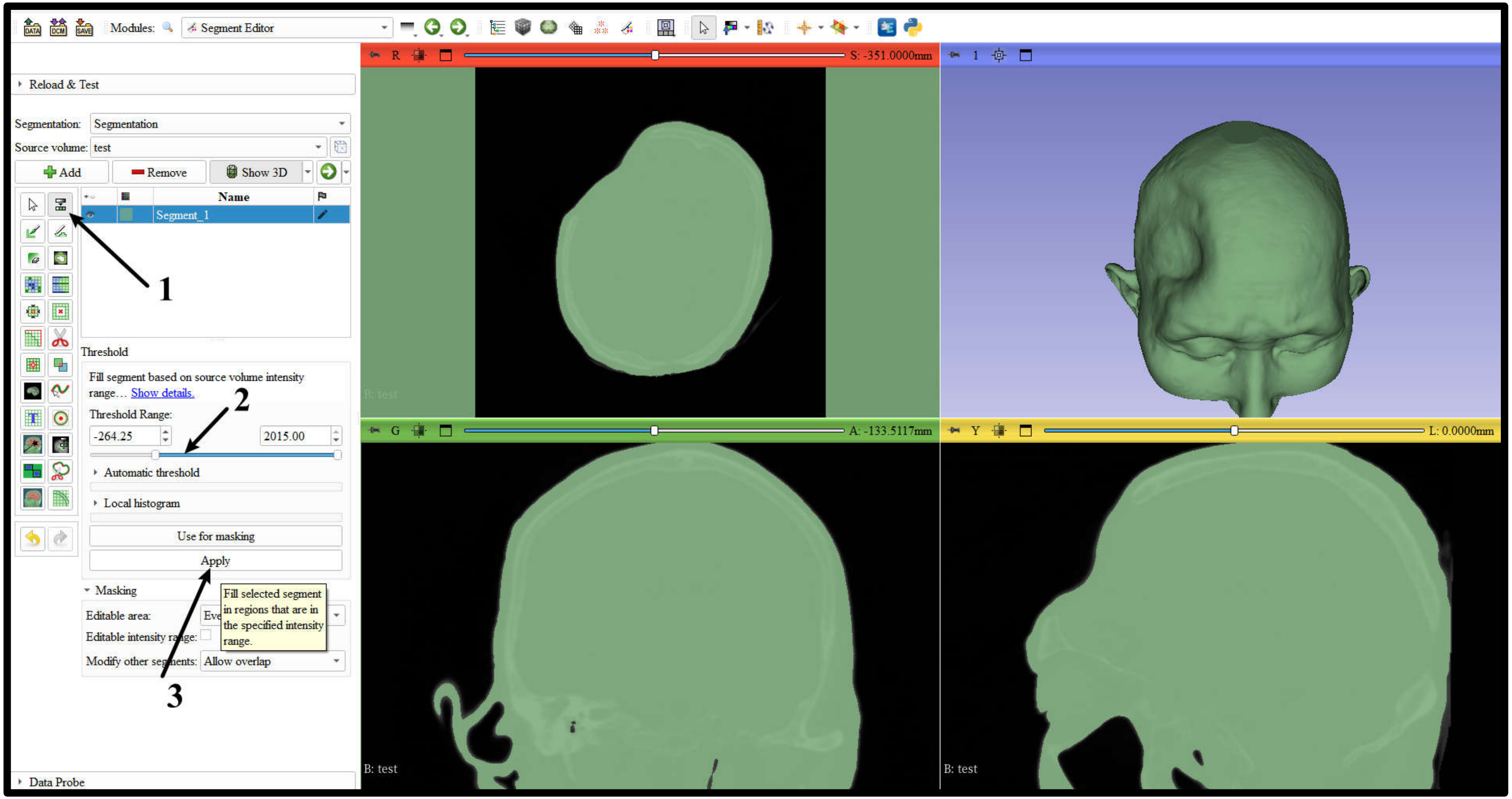

5. Mirror Alignment: Select the crosshair tool, then move the mouse near the crosshair lines to rotate and translate the view until the yellow slice view aligns with the midsagittal plane.

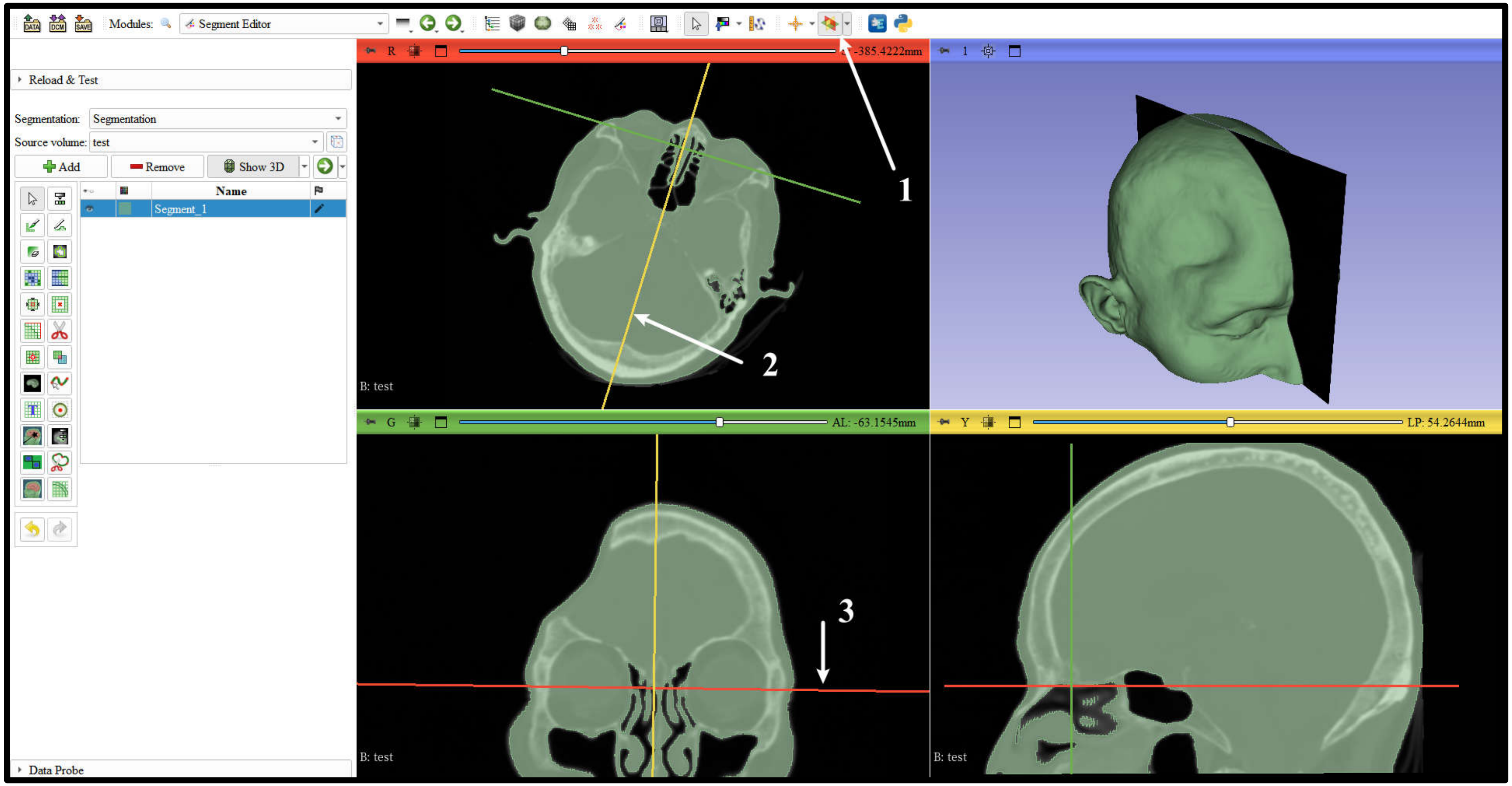

6. Remove the Defective Side: Select the *Scissors* tool and set the operation mode to *Negative* or *Positive*, then perform the cropping in the yellow slice view.

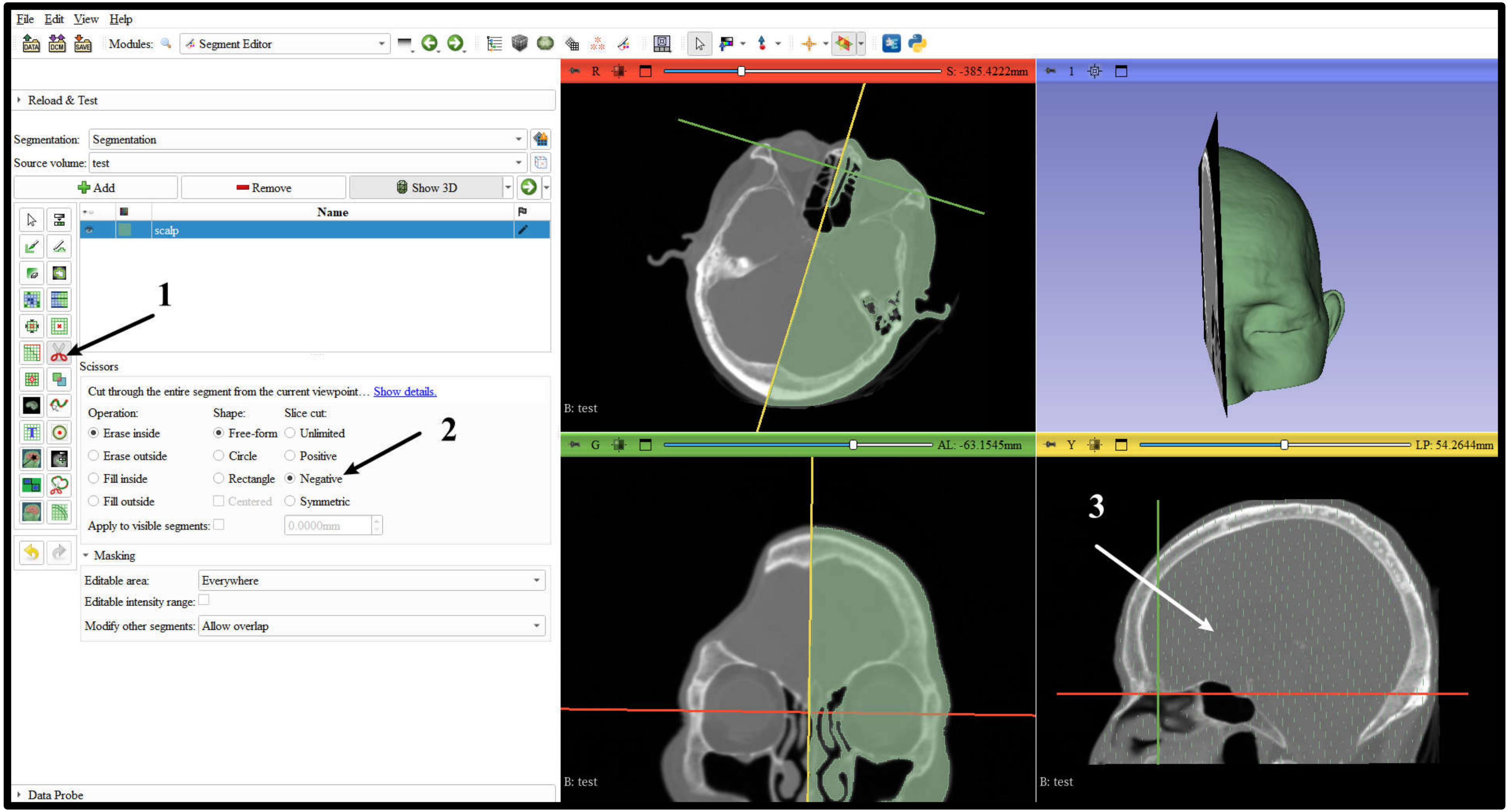

7. Create Mirror Model: Select the *Dynamic Modeler* module, then choose the *Mirror* tool. Set the *Model* and *Mirror plane*, and click *Apply*. If the initial mirror result is not satisfactory, you can fine-tune the crosshair position to adjust the mirror plane.

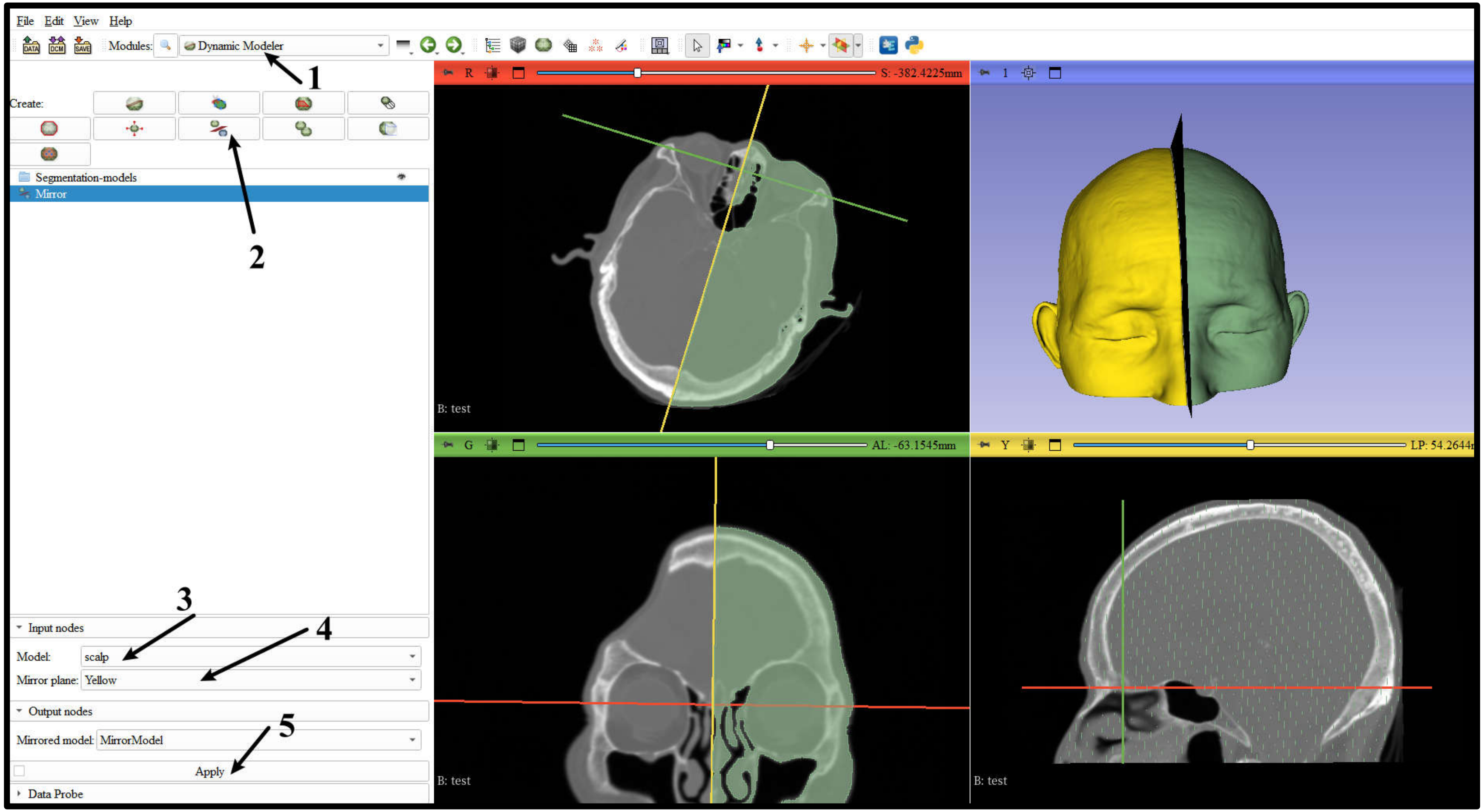

8. Reload Mirrored Model: Go to the *Data* module and drag the generated mirrored model into the *Segmentation* to convert it into a segment.

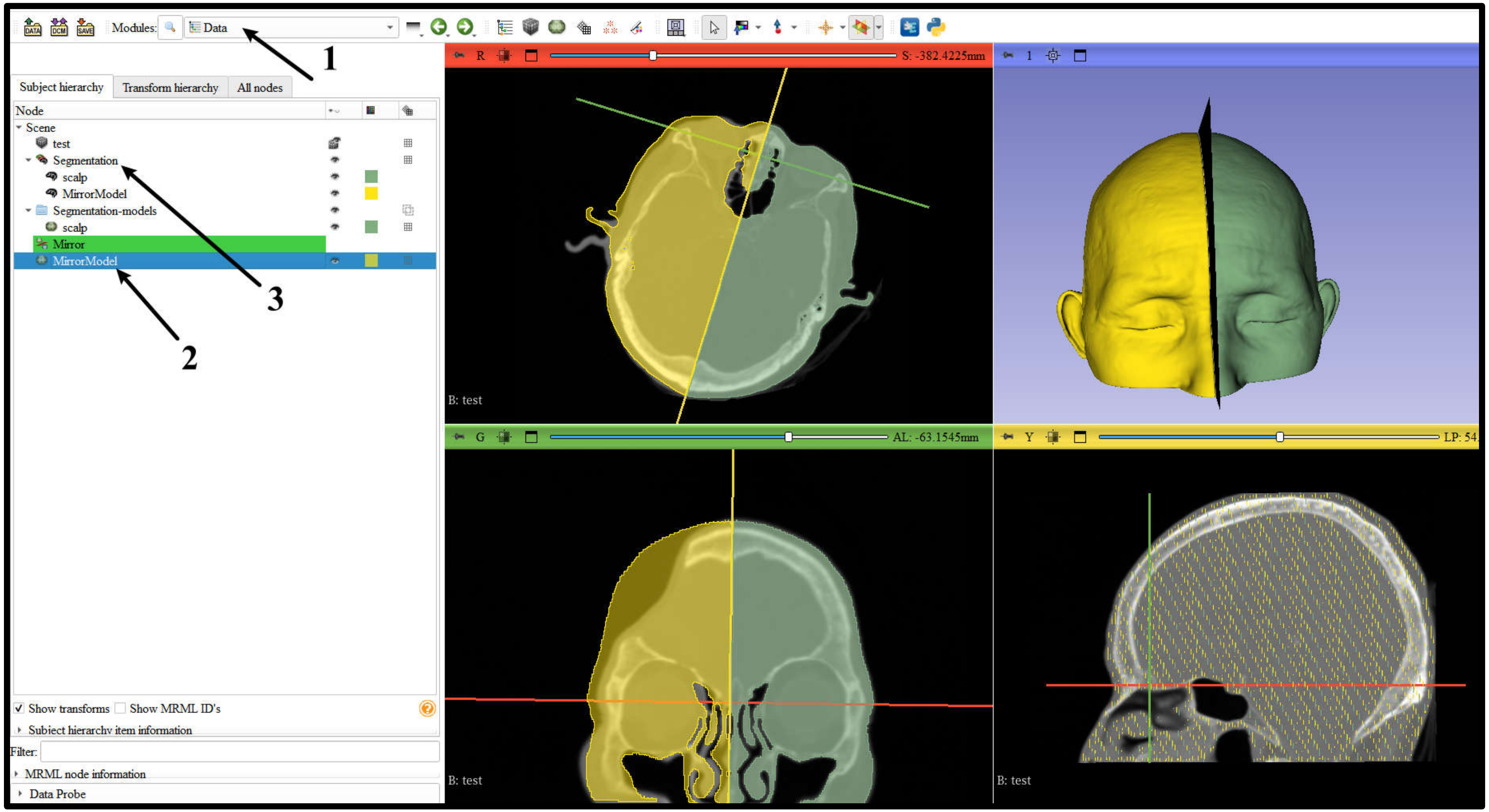

9. Merge Mirror Model: Select the *Logical Operators* tool, set the *Operation* to *Add*, then select both the original *scalp* and the *MirrorModel*.

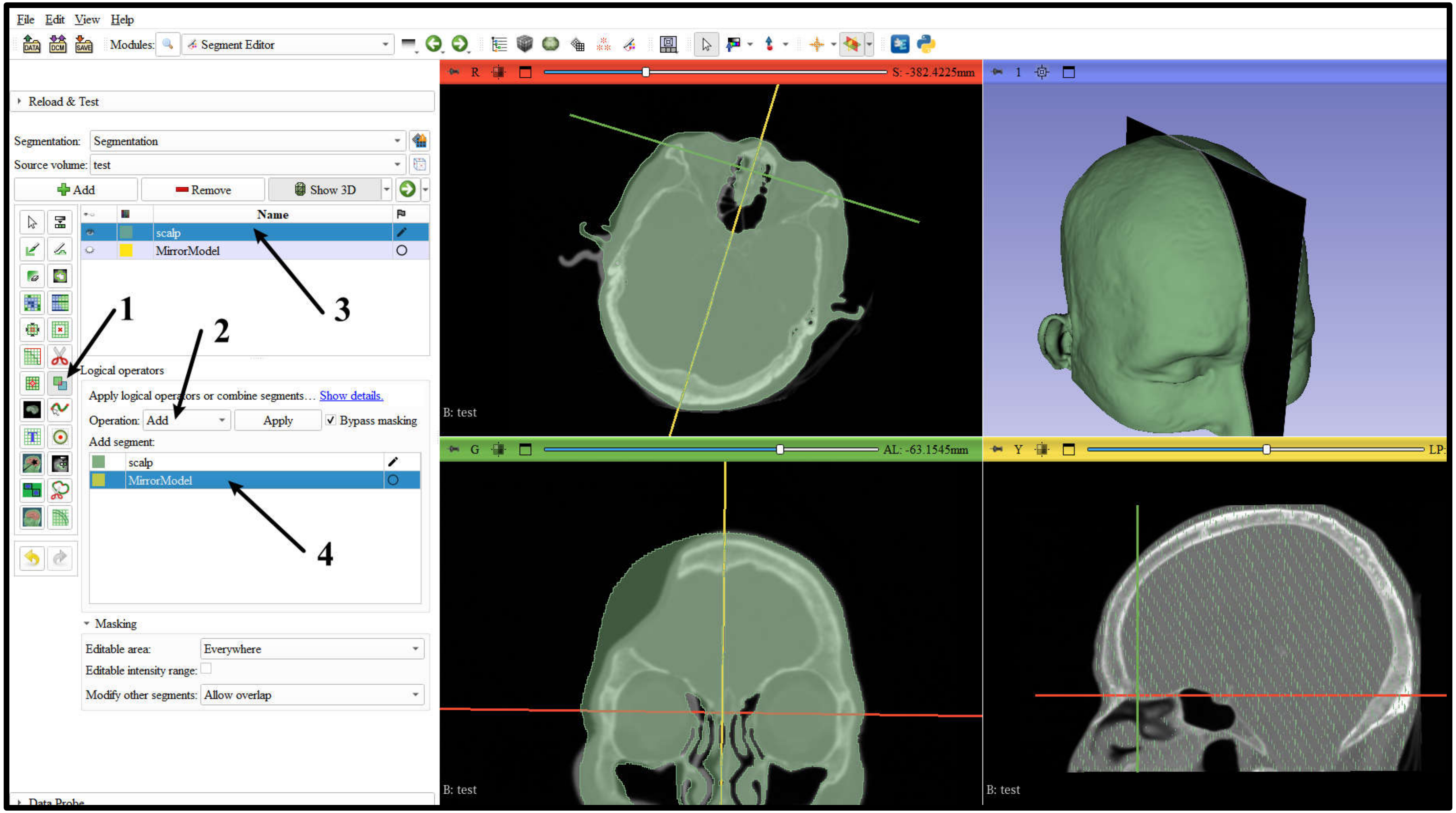

10. Hollow the Model: Select the *Hollow* tool, set the mode to *Inside surface*, specify a thickness of **2.5 mm**, and click *Apply*.

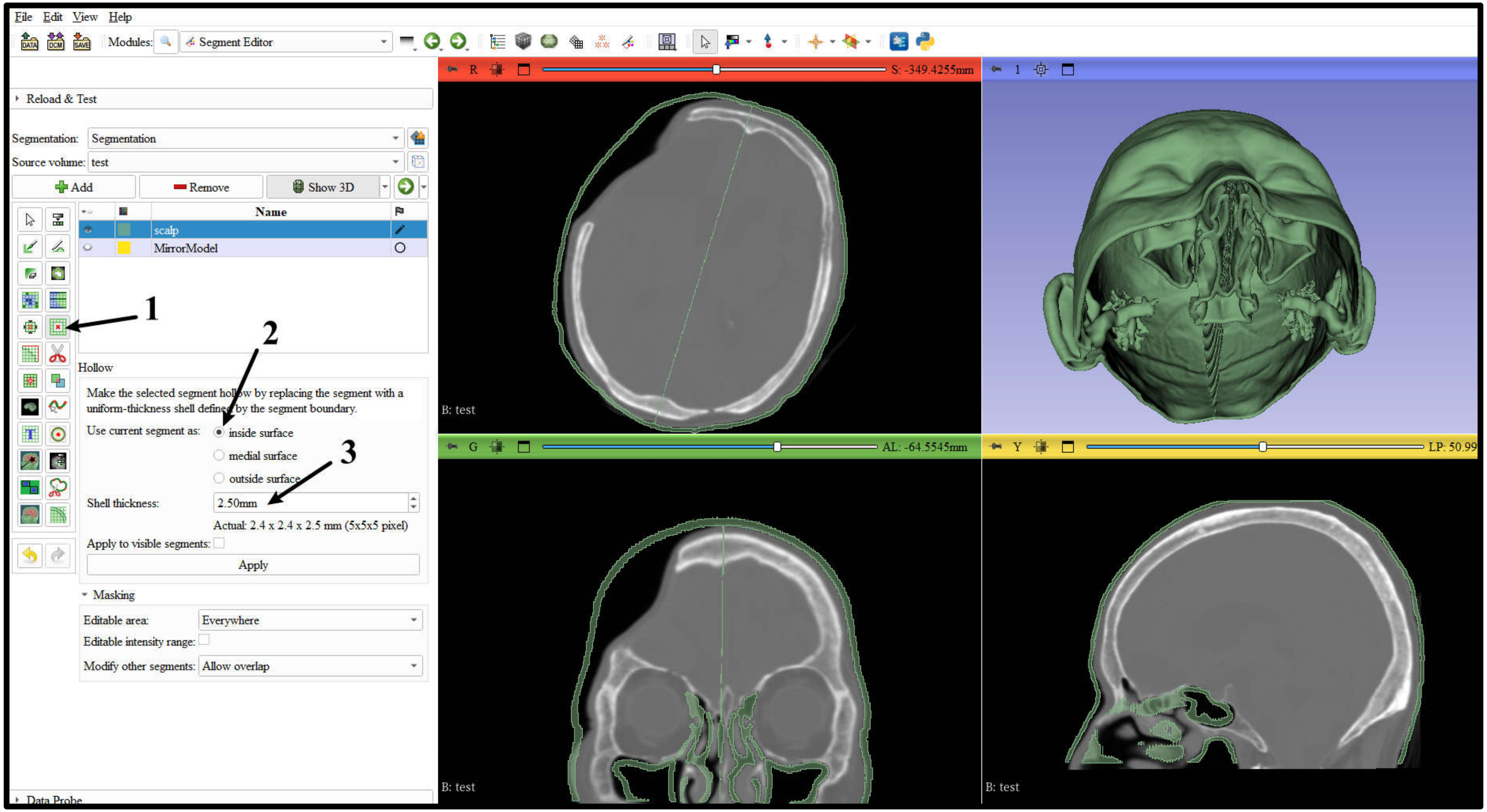

11. Model Trimming: Adjust the CT rendering settings, then use the *Scissors* tool to enlarge the defect area and create openings as needed.

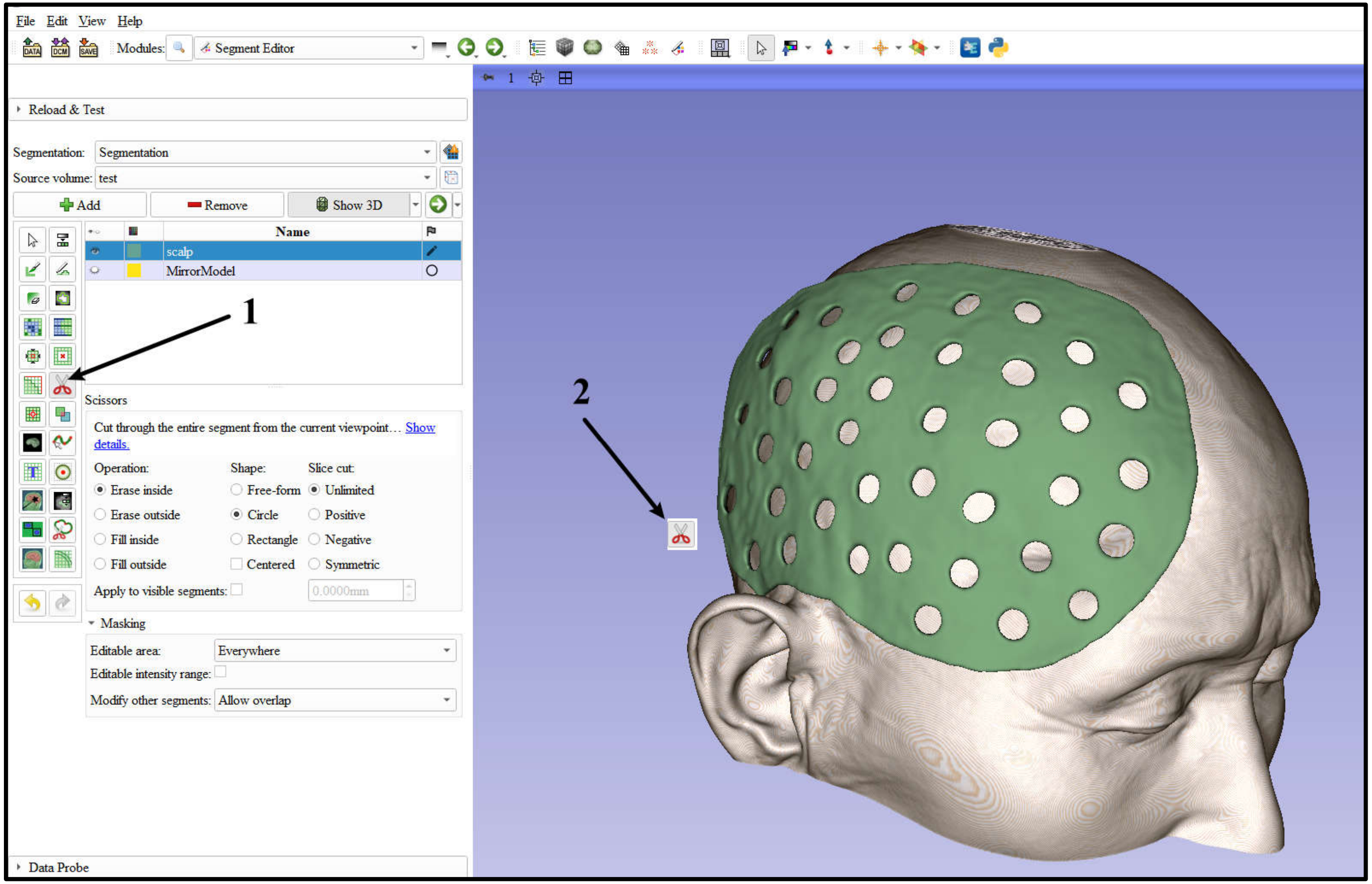

Supplement: Supplementary file 1 — Supplementary Material 1 [file 41205_2025_289_MOESM1_ESM.pdf]
